# Supplementary material for: Are carbon emissions trading and green financial instruments synergistic? -Comprehensive quantitative research based on content analysis
Source: PLoS One. 2024 Mar 7;19(3):e0298601. doi: 10.1371/journal.pone.0298601 (PMC10919601; doi:10.1371/journal.pone.0298601)
Supplement: S1 Data — (ZIP) [file pone.0298601.s001.zip › Data/地级市Z_F工作报告.zip!/╦─┤¿/┼╩╓a╗¿╩╨/└┤╫╘╣1⁄2╓┌║┼í╛┬φ┐╦╩2╛▌═°í┐_2014.docx]

各位代表：

　　现在，我代表市人民政府，向大会报告工作，请予审查，并请政协委员和列席同志提出意见。

　　一、2013年工作回顾

　　过去的一年，是我市发展历程中极不平凡而又特殊的一年。在省委、省政府和市委的坚强领导下，在市人大、市政协的监督支持下，市政府认真履行职能，紧紧依靠全市各族人民，攻坚克难，奋力拼搏，经济社会发展稳中有进、稳中向好，民生得到持续改善。全年实现地区生产总值800.88亿元，比上年（下同）增长10.7%，位居全省第八；完成全社会固定资产投资545.74亿元，增长17.8%；实现社会消费品零售总额219.33亿元，增长14.2%；实现全口径公共财政收入116.09亿元，其中，地方公共财政收入58.55亿元，增长2.4%；城镇居民人均可支配收入24906元，增长9.2%，农村居民人均纯收入9838元，增长12.7%；城镇登记失业率为3.5%；人口自然增长率为2.6％。；单位生产总值综合能耗预计下降3.3%。

　　经济结构调整取得积极进展。始终坚持做精一产、做强二产、做大三产的思路，持续推进产业升级和结构优化，关闭煤矿42家，淘汰了22户企业的落后产能，第一产业实现增加值27.88亿元，增长4.5%，第二产业实现增加值597.19亿元，增长11.7%，第三产业实现增加值175.81亿元，增长8%，三次产业对经济增长的贡献率分别为1.4%、83.2%和15.4%，民营经济增加值占GDP比重达46.1%，高新技术产业产值首破百亿、达110亿元。工业经济平稳较快增长。出台了稳定工业增长的系列措施，设立了企业应急转贷专项资金，累计周转资金12.92亿元，55家企业获得应急帮助。钒钛及相关产业实现产值175.9亿元，增长12.2%；机械制造业实现产值142.5亿元，增长18.9%。钒钛产业园区创建为四川省高新技术产业园区，六大工业园区实现规模以上工业总产值750亿元，增长14%。攀钢减亏61.7亿元，钢城集团名列2013年中国民营企业500强第175位和中国制造业500强第292位，十九冶利润增长11%。规模以上企业净增38户，总数达到376户，实现规模以上工业增加值452.9亿元，增长12.8%，位居全省第七。第三产业加速壮大。《中国阳光康养旅游城市发展规划》编制实施，“八大旅游项目群”加快建设，乡村旅游异军突起，重大旅游项目建设经验在全省推广，欢乐阳光节影响力逐步扩大，我市荣膺中国最具魅力的节庆城市，旅游总收入增长52.9%。我市成为全省首批现代物流业示范市，炳草岗商贸流通集聚区被列为四川省现代服务业集聚区，格里坪鑫铁工业物流园一期、米易青杠矿山服务物流中心等项目顺利竣工，快递业健康发展。城市商业网点规划修编完成，在全省同级市州中率先引进马来西亚金狮百盛国际知名连锁品牌和台湾太平洋大型连锁企业，消费带动和辐射能力增强。全市金融机构本外币各项存款余额达785.7亿元、贷款余额达611.58亿元，分别较年初增长3.5%和14.5%，存贷比位居全省第一。现代农业加快发展。积极推进国家现代农业示范区建设，实现农林牧渔业总产值48.1亿元，增长4.6%，其中晚熟芒果、早春蔬菜、优质烤烟分别实现产值5.19亿元、11.07亿元和4.15亿元，分别增长15.6%、7.7%和4%。创新品牌营销模式，在成都成功举办首次特色水果推介会。攀枝花块菌、盐边桑葚、米易苦瓜、米易山药被认定为国家地理标志保护产品。新增家庭农场367个、农民专业合作社148家。马鞍山水库、梅子箐水库（扩建）等项目进展顺利，整治病险水库20座，建成高标准农田2.3万亩，综合治理水土流失117.5平方公里。46个市级新村点完成年度建设任务。

　　投资拉动成效显著。《2013—2017年重大项目规划》编制完成，涵盖项目804个。实行“一个项目、一套班子、一套方案、一抓到底”的工作机制，强力推进重大项目。朵实10万吨锻铸件、密地尾矿回收利用、西宇生物一期、攀枝花学院2.1兆瓦太阳能屋顶光伏发电等重大项目竣工投产，富邦1000万件钒钛制动毂、瑞钢渣钢渣铁综合利用、观音岩和桐子林水电站、国省干线改扩建等项目推进加快，成昆铁路新线攀枝花段、缅气入攀、观音岩引水工程等项目开工建设，机场成功复航并增开至重庆、北京航线，丽攀高速公路攀枝花城区段建成通车，攀大（理）绕城高速公路招商成功。积极参加世界华商大会、西博会、科博会、农博会等活动，主动对接知名企业，新签约项目233个（其中投资额10亿元以上重大项目40个），履约项目372个，到位资金434.65亿元，增长6.4%。

　　城乡面貌有了新的变化。深入实施“五创联动”，加快推进68个攻坚项目，顺利通过省级森林城市、省级文明城市考核验收。启动编制全省首个市域城乡统筹发展规划，积极推进道路网络、城市风貌等专项规划编制。新密地大桥、龙密路、炳二区主干道等项目建成通车，倮果大桥、巴关河大桥等5座桥梁完成重建或除险加固，马坎污水处理厂及截污干管工程、花城大道老黑地至龙塘沟及连接线等项目有序推进，硬化县乡道路48.3公里，建成通乡柏油路（水泥路）64.9公里、通村公路257.84公里。建成6处地下人行过街通道，有效缓解了中心城区交通拥堵问题。花城新区建设提速，以“双十字”路架为支撑的重大项目群加快推进。跨时5年、共48个项目、惠及1.8万户群众的旧城改造正式启动，

　　瓜子坪商场老旧房屋改造等8个项目开工建设。小城镇建设成效显著，全省“两个现场会”在我市召开，红格镇、普威镇成为全省产城融合的典范。花城景观、沿江景观打造扎实推进，生态景观效应不断凸显。强化市城管委职能和监管力度，城市管理水平有效提升。

　　环境保护进一步加强。扎实开展国模创建，全面加强污染源和城乡环境综合治理，狠抓城市扬尘治理，深入推进重金属、危险废物和持久性有机污染物防治，主要污染物排放完成省政府下达的目标任务，预计单位工业增加值综合能耗下降6%。启动开展重点区域流域污染防治三年攻坚，完成年度攻坚项目36个，二氧化硫浓度下降25.6%，氮氧化物年均浓度下降7.5%，环境空气质量优良率达92.6%。切实加强饮用水水源地、地表和地下水系环境监管，水环境质量继续保持较好水平。

　　发展活力有效增强。经过多年努力，攀西国家级战略资源创新开发试验区（以下简称“试验区”）成功获批，钒钛资源创新开发上升为国家战略，三区两县成为核心区。率先推动试验区建设向“深水区”挺进，科学编制了一系列工作规划，积极争取上级支持，获得专项资金3.3亿元，在直购电试点、资源税改革试点、工矿废弃地复垦利用、设立国家级“攀枝花（钒钛）产业投资基金”等方面取得突破性进展，科技攻关、人才培养、项目集成等工作取得阶段性成效。全国唯一的跨省区产权交易机构西南联合产权交易所在我市组建分所，资源市场化配置能力有效提高。市属经营性国有资产进一步整合，户籍制度改革试点有序开展，食品药品监管机构改革基本完成，农村综合配套、人事制度、文化体制、医药卫生体制等各项改革取得新进展。加大简政放权力度，市级行政审批事项取消14项、下放2项。加快推进国家外贸转型升级专业型示范基地建设，实现外贸进出口总额1.87亿美元，东盟成为我市最大出口市场。国家知识产权示范城市创建工作扎实开展，第二届攀枝花钒钛（国际）论坛成功举办，重大关键技术攻关取得新成绩，“三大国家级创新平台”建立运行，科技进步获奖数量位居全省第二，新增国家高新技术企业16户，专利转化实现产值32.4亿元。

　　民生保障更加有力。圆满完成十大民生工程，总投资达39.99亿元，增长23.1%，民生支出占公共财政支出的64.7%。城镇新增就业1.77万人，失业人员再就业8015人，就业困难人员再就业2186人，藏区“9+3”学生初次就业率达100%。全市15万企业退休职工月均基本养老金达2063元，高出全省平均水平528元；新型农村合作医疗参保率稳定在99%以上。集医保卡、银行卡、公交卡等功能于一体的市民卡正式发行。在全省率先建成“12349”居家养老服务信息平台，面向全市18.3万老年人提供紧急救援、生活帮助、主动关爱和医疗服务。开工建设保障性安居工程6384套、竣工4189套，发放廉租住房补贴3440户，公租房对外来务工人员定向提供比例达90%。完成农村危房改造2728户，解决了4.6万农村人口的饮水安全问题。移民后扶整村推进示范片加快建设，“8+8”对口援边成效显著，建成产业路93公里，受益群众超过2万人。加强食品药品安全监管，公众饮食用药安全得到保障。居民消费价格指数上涨1.3%，比全国、全省平均指数低1.3和1.5个百分点。地震监测手段数字化改造顺利完成。按照省委、省政府要求，对口援助藏区凉山州木里县、灾后援建雅安市名山区等工作扎实推进、成效显著。

　　社会事业全面进步。各级各类教育协调发展，新建城乡公办幼儿园8所；五十一阳光家园小学建成投用，“两免一补”实现城乡全覆盖，三区顺利通过省政府义务教育均衡发展督导评估，在全省率先实行一师一校教师外出疗养制度；本科和重本上线率分别达42.5%、9.3%，均高于全省平均水平；3所中职学校成为国家中等职业教育改革发展示范学校建设单位；攀枝花学院学科建设迈上新台阶，四川机电职业技术学院被列为国家高技能人才培养基地。首批国家公共文化服务示范项目“大地书香新农村家园工程”顺利通过国家验收，三线建设博物馆开工建设，文物征集有序进行，文艺创作成果丰硕，文化产业加快发展。应急广播体系建设的“攀枝花模式”在全国推广，广电公共服务水平保持全省领先。县级公立医院全面取消药品加成，市中心医院第二住院大楼竣工投用，市中心医院医学影像中心建成省级重点专科，新增住院医师规范化培训基地8个，示范社区卫生服务中心数量在全省仅次于成都。国家卫生城市创建成果不断巩固，新增省级卫生乡镇4个、省级卫生村46个。全民健身计划深入实施，公共体育场馆免费开放79万人次，阳光体育产业加速发展。

　　社会治理创新得到强化。抓好本质安全型城市构建和全国综合减灾示范社区建设，深入开展安全生产专项整治，加大煤矿整顿力度，安全生产形势持续好转。积极开展社会稳定风险评估，有效畅通群众诉求渠道，“12345”市民热线办结率为99.5%，重点信访积案化解率达100%，网上信访办理率实现“四个100%”。积极开展“五规范五减少社区（村）”创建活动，试点社区（村）治安案件、可防性侵财案件发案率分别下降11.8%和8%。进一步完善社会治安整体联动防控体系，深入开展各类专项整治行动，群众安全感达95.2%。在全省率先普推“计生e通”，人口环境保持良好。积极维护民族团结，依法管理宗教事务，社会大局保持稳定。

　　此外，审计、人防、国安、出入境检验检疫、海关、气象、供销、档案、机关事务管理、住房公积金管理、地方志、邮政、外事、台务、侨务、妇女、儿童、残疾人等各项工作都取得了新成绩，国税、地税、工商、质监、烟草、通讯、石油、保险等驻攀单位也为全市经济社会平稳较快发展作出了新贡献。

　　各位代表，成绩来之不易，凝聚了大家的智慧和力量！这是市委坚强领导的结果，是市人大、市政协监督支持和军分区有力保障的结果，是全市上下开拓进取、主动作为的结果。在此，我代表市人民政府，向全市各族人民，向驻攀解放军、武警官兵和政法公安干警，向给予政府工作大力支持的人大代表、政协委员、各民主党派、工商联、无党派人士、人民团体和社会各界人士，向各位老领导、老同志，向来我市创业发展的建设者、投资者，表示衷心的感谢，并致以崇高的敬意！

　　总结一年的工作，我们清醒地看到，在全国、全省经济持续下行的压力传导下，我市长期积累的结构性矛盾与产能过剩、要素制约、煤矿整顿关闭等阶段性困难叠加交织，影响了地区生产总值、公共财政收入、城镇居民人均可支配收入等预期目标的实现。此外，我市发展中还存在不少矛盾和问题，主要表现在经济总量不大、产业结构不优、三产发展滞后、转型任务繁重、经济发展质量和效益不高、增长动力不足、投资大带动强的重大项目少、边远山区生产生活条件仍然较差等方面。从政府自身来说，效能建设仍需加强，少数工作人员还存在推诿扯皮、作风漂浮等现象。对这些问题，我们将高度重视，绝不以事艰而不为，绝不因任重而畏惧，采取有力措施，切实加快解决。

　　二、2014年主要任务

　　今年，政府工作的总体要求是：以党的十八大和十八届二中、三中全会精神为指导，深入贯彻落实中央和省委经济工作会议暨城镇化工作会议、市委九届六次全会精神，以科学发展为主题，以转变发展方式为主线，以新型城镇化为引领，以在全省率先全面建成小康社会为统揽，牢牢把握试验区建设、全国老工业基地调整改造和全省实施“三大发展战略”等重大机遇，坚持抢抓机遇、稳中求进、改革创新、加快发展，全力稳增长、抓改革、促转型、增效益、防风险、惠民生、创和谐，深入开展党的群众路线教育实践活动，坚定不移推进“三个加快建设”“三个走在全省前列”，集中力量打好“六大硬仗”，以优异成绩迎接建市50周年。

　　全市国民经济和社会发展主要预期目标为：地区生产总值确保增长10%，力争11%；全社会固定资产投资增长13%；公共财政收入增长7%；社会消费品零售总额增长12%；城镇居民人均可支配收入和农村居民人均纯收入均增长11%；城镇登记失业率控制在4%以内；人口自然增长率控制在4％。以内；单位生产总值综合能耗下降3%。

　　上述指标的确定，主要基于两个方面的原因：一是充分考虑发展大势和政策导向。当前，宏观经济形势依然严峻，下行压力仍然较大，经济增长由“高速”向“中高速”转换渐成共识，淡化增长、调整结构、突出改革已成全国全省工作主调。顺应大势，我市必须把经济增长的速度控制在合理范围内，以经济增长的主动适度减档为转方式、调结构留出必要空间。二是充分考虑发展实际和转型需要。“发展不足、发展不够、发展不充分”仍然是我市的最大实际，如果没有积极可行的发展速度，完成“十二五”规划、保障改善民生等都将难以实现。立足实际，我们必须既主动“抓紧转”，又坚持“加快赶”，在提质增效的前提下努力保持快于全国全省的增长速度，进一步做大总量，为在全省率先全面建成小康社会奠定坚实基础。

　　今年，要重点做好四个方面的工作：

　　（一）以提升质量和效益为核心，坚定不移稳增长、调结构，迈出转型发展新步伐

　　——力促经济平稳较快增长。强化投资关键作用，实行重大项目市长推进制。完成134个重大项目的年度任务，其中，开工建设攀钢钛业10万吨钛白粉、中国重汽矿用车组装线、天民钛业二期等58个重大项目，加快推进特利小五金产业孵化园、瑞钢渣钢渣铁综合利用、观音岩和桐子林水电站等49个重大项目，确保富邦1000万件钒钛制动毂、攀枝花Ⅱ220千伏输变电工程等27个项目竣工投产。继续实施“一企一策”，做好服务帮扶和梯次培育工作。有序推进煤矿复产，积极争取电价优惠，确保工业平稳运行。加快园区基础设施和服务平台建设，力争将钒钛产业园区创建为国家级高新技术产业开发区和国家级出口质量安全示范区。突出特色，错位发展，不断壮大县区经济，加快形成多点支撑发展的新格局。

　　——推动经济结构转型升级。坚持走新型工业化道路，确保规模以上工业增加值增长12%以上。推动小选矿、表外矿利用、钛白等企业兼并重组，探索“煤电冶”或“水电冶”集团发展模式。加快建设以攀昆公司为主的钛材深加工园，推动金沙纳米与中信集团深度合作，积极争取钒钛新材料在全省装备制造业中的应用推广，拓宽钒产业发展空间，延伸钛产业链，做大做强钒钛、机械制造、矿业等特色优势产业，努力化解过剩产能。大力发展太阳能、生物等新兴产业。加快发展现代服务业，确保服务业增加值增长8.5%。有序推进阿署达花舞人间、普达阳光国际康养度假区等项目群建设，加快打造庄上等旅游新村，确保红格温泉旅游度假区创建为国家级旅游度假区。加快推进密地现代商贸物流园、攀青煤炭物流中心、银泰城市综合体等重点项目，做好现代物流领域标准化试点示范工作。大力发展阳光康养、物流快递、社区服务等生活性服务业，着力推进生产性服务业与制造业融合发展，力争成为全省现代服务业改革试点市。大力发展现代农业，确保农林牧渔业总产值增长4%。以特色优势产业为重点，以门里集团现代农业综合产业园等项目为带动，加大国家现代农业示范区建设力度，加快发展外向农业、品牌农业、设施农业和观光休闲农业。扎实推进马鞍山水库、观音岩引水工程、梅子箐水库（扩建）等重点水利项目，建设高标准农田2万亩，新建万亩示范区3个。积极培育农业产业化龙头企业、大户集群和新型农业经营主体，强化农产品质量安全监管，构建内外相连、产销相见的农产品营销网络，促进农业增效、农民增收。扩大农业保险范围，提升抗灾能力。

　　——加快建设攀西试验区主体功能区。先行先试，探索借鉴“负面清单”等管理模式，力争在产业、交通、投资、财税、土地、环保、供电等重点领域和关键环节实现更大突破，加快把我市建设成为钒钛产品生产中心、质检中心、研发中心和交易中心。做好项目谋划、储备和包装，力争更多项目进入国家和省上的总盘子。进一步完善科技攻关、财税支持等工作方案，用好用活试验区建设专项资金，举办钒钛产业博览会等活动，吸引带动更多资本和研发机构参与钒钛资源创新开发。

　　（二）以改革开放为引擎，坚定不移抓创新、破难题，增添转型发展新动力

　　——全面深化各项改革。继续深化国资国企改革，推动市属国有资产布局结构调整。支持非公有制经济健康发展，鼓励和引导符合条件的个体工商大户转型升级。稳妥做好农村承包土地确权颁证试点工作，探索建立农村土地产权交易中心。稳步推进户籍制度改革，优先引导在城镇有稳定就业和住所的农村转移人口落户。积极推进事业单位分类改革，进一步深化财政、科技、教育、文化、卫生等体制改革。

　　——不断扩大对外开放。创新方式招大引强，着力引进产业链构建中的关键企业和带动力强的品牌企业，实现招商引资到位资金450亿元。以国家外贸转型升级专业型示范基地建设为抓手，鼓励攀钢、东方钛业、白云铸造、十九冶等外贸领军企业做大做强，支持天成丝绸、新中钛科技、海峰鑫化工等外贸成长型企业壮大规模，助推兴中钛业、恒源石化等潜力企业实现自营出口。鼓励开展国际工程承包和劳务合作，积极推动境外投资。围绕试验区建设，强化与凉山、雅安等市州的战略合作，推动建立市州联席会议、重大项目会商等工作机制，力争在打破行政壁垒、优化区域产业布局等方面取得新成效。

　　——提升科技创新能力。依托钒钛资源综合利用国家重点实验室、国家钒钛检测重点实验室和四川钒钛产业技术研究院等科研平台，鼓励攀钢、龙蟒、钢城集团等企业采取市场运作方式，协同开展关键瓶颈技术攻关。办好试验区科技专项对接会，做好重大科技项目招投标工作。加快科技成果转化及产业化步伐，积极打造国家级太阳能应用研发基地，新培育国家高新技术企业8户，实现高新技术产业产值130亿元，争取成为国家创新型城市试点，确保创建为国家知识产权示范城市。

　　——努力破解发展难题。探索“财政支持金融、金融扶持产业”发展模式，不断拓宽筹融资渠道。大力推进老工业基地调整改造、“城市矿产”建设试点和钒钛磁铁矿整装勘查工作，建立健全矿产资源开发进入和退出机制。加快推进成昆铁路新线攀枝花段、攀大（理）绕城高速公路等项目建设，做好丽攀昭遵铁路、攀盐（源）高速公路等项目前期工作。全面完成国省干线三年攻坚任务，力争开通至广州、上海等航线。切实抓好节能减排，扎实推进重大节能工程和污染源限期治理项目，淘汰一批落后产能。创新人才政策，加大对高层次、领军型人才的引进和培养力度，加快建设国家钒钛产业高技能人才培养基地。

　　（三）以新型城镇化建设为抓手，坚定不移统城乡、增内涵，展现转型发展新成效

　　——大力推进城乡建设。科学统筹城乡全域规划，加强规划管理和执行。强力推进以“双十字”路架为中心的花城新区基础设施和重大项目建设。加快实施旧城改造，按计划推进创业路、华山片区原建二公司等老旧房屋改造项目。推广红格小城镇建设经验，更加注重产城融合，积极打造一批独具特色、风格各异的新型城镇。不断优化市域路网，加快推进干坝塘至鱼塘高速路出口连接线等项目，开工建设红格西环线一期、地龙箐至金沙江大道连接线立交工程等项目。加快构建贫困地区交通大环线，力争提前实现100%的行政村通柏油路（水泥路）。继续抓好省级新农村建设成片推进示范项目和新村点建设，确保通过省级验收。

　　——不断改善人居环境。纵深推进“五创联动”，确保完成年度任务。继续抓好沿江景观和花城景观打造，积极推进沿江休闲、娱乐等设施建设，加快把金沙江中心城区段两岸打造成独具特色、风景亮丽的生态景观带和休闲娱乐带。结合环境空气质量新标准的实施，突出抓好扬尘专项治理，深入开展以“三片一线一企”为重点的区域流域污染综合整治，督促推进攀钢新1号烧结机脱硫系统优化改造等项目，促进环境空气质量明显提升。加大饮用水源一级保护区隔离带建设力度，确保饮水安全。深入实施市区环城景观林建设和天保二期工程，争取启动金沙江干热河谷区生态恢复和治理试点工作。强化城市管理，建立完善覆盖城区和重点乡镇的数字化城管体系，继续抓好城乡环境综合治理。

　　——着力提升文化软实力。积极倡导社会主义核心价值观，大力实施公民道德建设工程，有效提升市民文明程度。加快建设区域文化高地，高效推进三线建设博物馆、市中心图书馆等标志性文化设施建设，规划建设一批特色鲜明、内涵丰富的街区街景，办好四川省第七届少数民族艺术节。高标准构建以县级为枢纽、乡级为骨干、村级为基础的基层公共文化服务体系。加快发展文化产业，有序推进国家级苴却砚文化园区等项目。以迎接建市50周年为契机，深度凝炼三线文化精髓，传承弘扬攀枝花精神，增强市民的归属感和自豪感。

　　（四）以民需民盼为导向，坚定不移惠民生、促和谐，凝聚转型发展新合力

　　巩固创业型城市创建成果，确保就业总量稳中有增、就业质量不断提升。积极构建一体化的城乡居民社会保障体系，调整城乡居民养老保险缴费档次和补贴标准，提高医疗保险及大病报销水平。围绕区域教育高地建设，坚持教育优先发展，抓好中小学信息化和标准化建设，着力促进城乡教育均衡发展，努力提高教育教学质量。以打造区域医疗卫生高地为抓手，加快花城新区医院、市妇幼保健院（迁建）等项目建设，完善卫生应急指挥决策系统，不断提升医疗服务水平和公共卫生保障能力。认真落实《全民健身条例》，组团参加第十二届省运会，大力发展阳光体育产业，努力打造亚高原训练和冬季训练两大品牌。深入开展对口帮扶，实施“五大扶贫工程”，促进边远民族地区加快发展。强化食品药品安全监管，确保“舌尖上的安全”。加强价格监管，保持物价平稳。畅通诉求表达渠道，推动信访积案化解，维护群众合法权益。强化隐患排查治理，坚决遏制重特大安全事故发生。加强社会治安综合防控，不断提升群众安全感。认真落实党的民族和宗教政策，巩固各民族团结奋斗、繁荣发展的良好局面。按要求实施“单独两孩”政策。切实抓好全国和谐社区建设示范城市创建工作。进一步做好国防动员、民兵预备役和“双拥”工作。扎实开展第三次经济普查。重视并做好人防、国安、出入境检验检疫、海关、气象、档案、防震减灾、地方志、外事、台务、侨务、妇女、儿童、残疾人等工作。

　　各位代表，今年，市政府将切实开展“走基层、解难题、办实事、惠民生”活动，继续加大投入力度，深入实施民生工程，为老百姓办好18件实事：硬化县乡道路160公里、村道60公里；建设公路安全护栏330公里；新增（更新）一批公交车辆；建设保障性安居工程6730套；新建一批人行过街设施；对1.8万名农村义务教育阶段经济困难寄宿学生补助生活费；减免1.94万名贫困家庭及民族地区家庭幼儿保教费；免除1.29万名中职学校学生学费；资助普通高中家庭经济困难学生6700人、中职学校家庭经济困难学生7400人；城镇新增就业1.6万人、失业人员再就业5000人、就业困难人员再就业1200人；建设村卫生室14个；完成8个贫困村整村推进任务，加快建设彝家新村；完成“8+8”对口援边投资2400万元以上；解决8.038万农村人口的饮水安全问题；建设城乡社区日间照料中心46个；资助5789名重度残疾人护理费用；新增78个行政村通宽带；新建金海农贸市场。

　　三、政府自身建设

　　加强政府自身建设，是做好工作、推动发展的前提和基础。2013年，我们以“抓落实”为核心，坚决执行中央“八项规定”和省、市“十项规定”，深入开展“实现伟大中国梦、建设美丽繁荣和谐四川”及“三大主题”教育活动，全面加强自身建设，政府执行力、创新力、公信力和应对复杂局势、驾驭发展全局的能力有了新提升。新的一年，面对深化改革和转型发展的重任，我们将时刻牢记“空谈误国、实干兴邦”，夙夜在公，勤勉工作，坚定发展信心，营造发展氛围，创新发展举措，保持发展态势，着力打造人民满意的法治政府、廉洁政府和服务型政府，以政府的“辛苦指数”提升城市的“发展指数”和市民的“幸福指数”。

　　——抓改革，加快转变政府职能。紧紧围绕市场在资源配置中的决定性作用来转变政府职能，处理好政府与市场、政府与社会的关系。进一步深化行政审批制度改革，强化审核事项后续监管，推进工商登记制度改革，探索建立省、市、县（区）三级联动的并联审批机制，逐步推行“联合审办、超时默认、缺席默认、勘查‘一车制’”。调整优化政务服务中心进驻项目，进一步简化办事流程。统筹用好有限的财政资金，最大限度发挥财政资金的导向作用。积极探索在筹融资、资源有偿使用等方面的创新和改革，有效防范和化解政府性债务风险。

　　——抓法治，提高依法行政能力。始终坚持以法治思维和法治方式履行政府职能职责，妥善解决各种矛盾和问题。自觉接受人大、政协及社会各界的监督，认真办理人大代表建议和政协提案，广泛听取社会各界意见建议。进一步健全行政决策机制，规范行使行政执法自由裁量权，落实行政执法责任制，营造公平开放的市场经济法治环境。深入开展“六五”普法，不断提升市民法律素质。

　　——抓作风，加强政府廉政建设。按照“照镜子、正衣冠、洗洗澡、治治病”的总要求，认真开展党的群众路线教育实践活动，确保活动出亮点、见实效。探索建立长效机制，巩固“四风”整治成果。进一步强化服务意识，使“马上办、现场办”“你有事、我加班”成为常态。严格执行《党政机关厉行节约反对浪费条例》等规定，细化操作规程，严控三公经费，严查违规行为。积极倡导敢于负责、勇于担当、勤于落实、善于突破的工作作风，严厉查处“慵懒散”及行政不作为、慢作为、乱作为等问题。加强惩防体系和廉政风险防控机制建设，保持惩治腐败的高压态势，及时有效解决损害群众利益的突出问题。加大审计监督力度，支持执纪执法部门依法开展工作。

　　——抓落实，确保完成工作任务。全面推进战略思路的方案化、项目化、具体化，打造决策、执行、监督、考评、奖惩一体化的“落实链条”，到一线、到现场去解决难题、推动工作。着力打破少数干部遇事先找“教条”、办事先看“惯例”、干事先想“常规”的思维，对既定事项雷厉风行抓落实，对急难险重任务勇于担当抓落实，对重大项目和民生实事定责问效抓落实。大力精简会议活动，大幅减少文件简报，腾出更多的时间和精力促发展、惠民生。

　　各位代表，困难和挑战考验着我们，责任和使命激励着我们！让我们在中共攀枝花市委的坚强领导下，同心同德，真抓实干，开拓进取，为把攀枝花建设得更加美丽、为让攀枝花人民生活得更加幸福而努力奋斗！
